# Current concepts in total knee arthroplasty: Rotating hinge prostheses

**DOI:** 10.1051/sicotj/2025010

**Published:** 2025-03-19

**Authors:** Tiffany Carol Oliver, Babar Kayani, Tianyi David Luo, Hugo Humphries, Fares Sami Haddad

**Affiliations:** 1 University College Hospital 235 Euston Road, Bloomsbury London NW1 2BU United Kingdom; 2 Princess Grace Hospital 42-52 Nottingham Place, Marylebone London W1U 5NY United Kingdom

**Keywords:** Arthroplasty, Rotating hinged knee, Knee implants, Knee replacement, Implant survivorship

## Abstract

This narrative review evaluates the purpose and functionality of rotating hinged total knee arthroplasty (RHTKA). The main indications for an RHTKA are poor bone stock, soft tissue compromise, gross instability, and periprosthetic fractures. Studies have shown that an RHTKA may be used in both the primary and revision scenarios to improve the range of motion and functional outcomes. Radiostereometric analysis has shown that some RHTKA designs are associated with early femoral component micromotion, but this has not translated to increased failure or revision rates. Implant survivorship with a modern RHTKA is comparable to a condylar-constrained TKA at mid-term follow-up. The most common complications associated with RHTKA are aseptic loosening, periprosthetic joint infection, stiffness and periprosthetic fractures.

## Introduction

The hinged prosthesis in total knee arthroplasty (TKA) was first introduced as a surgical implant made from an acrylic resin in 1951 by Walldius [1]. Following this, further first-generation hinged prostheses were created using metallic components. This included Shier’s stainless-steel hinged knee in 1953, Young’s Vitallium hinged knee in 1958, Guepar’s chrome-cobalt-molybdenum alloy hinged knee and Stanmore’s acrylic polymer with nylon axle hinged knee in 1969. These designs allowed only flexion-extension motion with one degree of freedom and relied directly on the implants to support the patient’s body weight [2]. The multi-directional forces across these implants led to early bearing wear and premature component loosening [2]. Second-generation hinged prostheses were developed to provide additional axial rotation with two degrees of freedom and included a polyethylene insert to minimise bearing wear. These were also prone to premature component loosening due to the high sheer forces at the bone-implant interface, altered knee biomechanics and patella maltracking [2]. Further design modifications have led to the development of third-generation hinge components, which allow for rotational movements of the implant [2]. These theoretically provide a more congruent articulation, decreasing implant wear and reducing inter-facial stresses. Modular segments have also been incorporated into modern hinge knee implant designs, allowing larger intramedullary bone defects to be filled. Despite these design features, concerns remain over implant survivorship, functional outcomes and complications following the use of hinge knee prostheses.

This narrative review provides an overview of the indications and complications of rotating hinged total knee replacement (RHTKA) and aims to assist surgeons in decision-making regarding the use of these prostheses.

## Indications for RHTKA implants

Patients with severe deformities, extensive bone loss, or ligamentous deficiency may require increased levels of constraint to optimise stability following TKA [2, 3]. In primary TKA, hinged implants may be indicated in patients with collateral ligamentous insufficiency, distal femoral or proximal tibial osseous defects, instability, knee extensor mechanism deficit, severe rheumatoid arthritis, severe varus/valgus deformities, Charcot arthropathy or oncological reconstruction [2, 4, 5]. In revision TKA, hinged implants may also be used for periprosthetic fractures [4, 6–8] and septic revisions with significant bone loss and soft tissue compromise [9].

## Range of motion and functional outcomes

RHTKA prostheses have been associated with an increased range of motion (ROM) and improved functional outcomes following primary and revision TKA. Yeroushalmi et al. showed an average range of motion (ROM) increase of 9 degrees compared with pre-operatively for 18 complex primary and 129 revision patients who had an RHTKA [10]. In a systematic review, Stroobant et al. identified 1131 patients who had revision surgery with a RHTKA and showed a mean ROM improvement from 69.1 to 104.1 degrees [11]. When comparing RHTKA between the primary and revision settings, studies have shown a non-statistically significant difference in final ROM [6, 12]. Collectively, these results show net improvement in ROM gain when using an RHTKA, with no significant difference in the ROM improvement when used for either the primary or revision setting. Neuhaus et al. [6] reviewed outcomes in 98 patients undergoing primary TKA and 22 patients undergoing revision TKA with the hinged EnduRo prosthesis (Aesculap AG, Tuttlingen, Germany). They reported improvements in the ROM using a goniometer for measurement (108.7°–117.9° in primary RHTKA and 110.7°–114.6° in revision RHTKA), knee society scores (kKSS) (35–89.5 for primary RHTKA and 37.5–78.1 for revision RHTKA) and functional knee society scores(fKSS) values (57.3 and 56.7–73.5 and 67.8 for primary RHTKA and revision RHTKA respectively) at five years follow-up. The primary RHTKA group improved their functional outcomes more than the revision group at a five-year follow-up. Similarly, Efe et al. [12] reported improvements in functional outcomes using hinged components in primary and revision TKA, though statistical differences were not found between primary and revision cases at five-year follow-up (Table 1).

**Table 1:** Shows the main data and results from the included studies in this narrative review.

Study title, Authors, Journal, Year published	Number of patients + follow-up	Indications	Functional outcomes (pre-op mean compared with post op)	Implant survivorship	Complications
**The use of a modular rotating hinge component in salvage revision total knee arthroplasty**	16 revision knees	‐ Aseptic loosening (8) ‐ Loosening and bone loss associated with chronic extensor mechanism disruption (2) ‐ Component instability (3) ‐ Fracture (1)	‐ KSCS 41–131 ‐ ROM 78°–93°	N/A	‐ Intraoperative fracture (1) ‐ Patella subluxation requiring revision (1) ‐ Mechanical failure of implant (1) ‐ Partial peroneal palsy (1)
Robert L. Barrack, MD, Thomas R. Lyons, MD, Robert Q. Ingraham, MD, Jeremy C. Johnson, MD	Mean follow-up of 51 months (2–6 years)				
The Journal of Arthroplasty					
2000					
**Salvage revision total knee replacement using the Endo-Model® rotating hinge prosthesis**	51 revision cases	‐ Infection (23) ‐ Aseptic loosening (23) ‐ Implant failure (3) ‐ Stiffness (1) ‐ Peri-prosthetic fracture (1)	‐ HSS 35.9–72.17 ‐ ROM 89.9° at one year f/u	N/A	‐ Amputation (2)
N.R. Pradhan, L. Bale, P. Kay, M.L. Porter	Mean follow-up of 4 years (range 2–6 years)				
The Knee					
2004					
**Rotating hinged total knee replacement: Use with caution**	44 patients	‐ Massive bone loss secondary to failed arthroplasty (27) ‐ Instability (10) ‐ Periprosthetic fracture (4) ‐ Comminuted distal femoral fracture (3)	‐ KSCS 28.9–73.5 ‐ KSFS 40–43 ‐ SF-36 physical: 28.3–41.1 ‐ SF-36 mental: 54.5–59.4	79.6% at one year and 68.2% at five years	Revisions ‐ Aseptic loosening (4) ‐ PJI (2) ‐ Periprosthetic fracture (1)Reoperations total ‐ Haematoma (8) ‐ PJI (3) ‐ Excision of retained cement (1)
Aidin Eslam Pour^1^, Javad Parvizi, Nicholas Slenker, James J Purtill, Peter F Sharkey	Minimum 2 year follow-up				
Bone and Joint Journal					
2007					
**Complications following rotating hinge Endo-Modell (Link** ^**®**^ **) knee arthroplasty**	Primary (52)	Primary ‐ OA with ligament laxity or significant malalignment (37) ‐ Tumour (1) ‐ Post traumatic arthritis (8) ‐ Rheumatoid arthritis (6)Revision ‐ Infection (9) ‐ Aseptic loosening (24)	NA	89.4% at three years	‐ Cement shock leading to death (1) ‐ PJI requiring joint lavage (2) ‐ CPN lesions partially resolving (2) ‐ Acute ischaemia requiring arterial surgery (1) ‐ PJI requiring revision components (2) ‐ PJI requiring arthrodesis (2) ‐ PJI treated with DAIR (3) ‐ Aseptic loosening (3) ‐ Patella complications (4)
B. Guenoun ^a^, L. Latargez ^a^, M. Freslon ^a^, G. Defossez ^b^, N. Salas ^a^, L.-E. Gayet	Revision (33)				
Orthopaedics & Traumatology: Surgery & Research	Mean follow-up 36 months				
2009					
**Mid-term results after implantation of rotating-hinge knee prostheses: Primary *versus* revision**	21 primary	Primary: ‐ Severe bone loss ‐ Ligamentous instability ‐ Malalignment ‐ Fracture ‐ Ankylosis ‐ InfectionRevision ‐ Infection ‐ Aseptic loosening ‐ Instability ‐ Periprosthetic fractures ‐ Malimplantation ‐ Poor ROM ‐ Progressive arthrosis	Primary ‐ KSCS 82 ‐ KSFS 58 ‐ ROM 88Revision ‐ KSCS 80 ‐ KSFS 47 ‐ ROM 89	Survival rates at final follow-up ‐ Primary 95% ‐ Revision 76%	Primary ‐ Recurrent dislocation (1) ‐ Periprosthetic Fracture (1) ‐ Infection (1)Revision ‐ Infection (8) ‐ Aseptic loosening (2) ‐ Inlay breakage (1) ‐ Periprosthetic fracture (1) ‐ Chronic pain (1) ‐ Septic loosening (1) ‐ Recurrent dislocation (1)
Turgay Efe^1^, Philip P Roessler^1^, Thomas J Heyse^1^, Carsten Hauk^2^, Caroline Pahrmann^3^, Alan Getgood^4^, Jan Schmitt	28 revision				
Orthopaedic Reviews	Mean follow-up 56 months				
2012					
**Rotating-hinge total knee for revision total knee arthroplasty**	31 Revisions	‐ Aseptic loosening (23) ‐ Septic loosening (4) ‐ Instability (3) ‐ Wear (1)	‐ HSS score 65.5–88.4 ‐ ROM 90.9–114.4	N/A	‐ Revisions for progressive radiolucent lines (2) ‐ Complications (10)
Alessandro Bistolfi^1^, Giuseppe Massazza, Federica Rosso, Maurizio Crova	Average follow-up 60.3 months (range, 32–100 months)				
Orthopaedics					
2012					
**Constraint choice in revision knee arthroplasty**	67 revisions to:PS knee (7)CCK (35)RHK (18)	‐ Ligament absence/disruption ‐ Type 2 or 3 AORI bone loss	Combined median results of all 3 implants ‐ IKS clinical score 41 to 81 ‐ IKS function score 21.5 to 79 ‐ HSS score 34 to 83.5 ‐ ROM 74 to 121	N/A	Hinged knee rerevisions total ‐ Persistent pain (1) ‐ Aseptic loosening (1)
Michele Vasso^1,✉^, Philippe Beaufils^2^, Alfredo Schiavone Panni^1^	Median follow-up 9 years (range, 4–12).				
International Orthopaedics					
2013					
**Revision knee arthroplasty for bone loss: Choosing the right degree of constraint**	94 revisions	Bone defect with AORI grade II or III bone loss	AORI II revision for infection increase of mean score: ‐ KSS by 47.2 ‐ KSFS by 53.3 ‐ Modified KSS by 31.6AORI III septic revision increase in mean score: ‐ KSCS by 28.7 ‐ KSFS by 11.5 ‐ Modified KSS by 23.1	NA	AORI II infections: ‐ Aseptic revisions (4) ‐ Septic revisions there were (3)AORI III infections: ‐ Aseptic (6) ‐ Septic (8)Other: ‐ Extensor mechanism failure (2) ‐ Aseptic loosening (1)
Chao Shen, MD, Paul M. Lichstein, MD, MS, Matthew S. Austin, MD, Peter F. Sharkey, MD, Javad Parvizi, MD, FRCS					
The Journal of Arthroplasty					
2013					
**Mid-term survival following primary hinged total knee replacement is good irrespective of the indication for surgery**	964 primary cases	‐ OA (686) ‐ Inflammatory arthropathy (95) ‐ Post-traumatic arthritis (81)	N/A	5 year survival rate of 96.8%	Revision (20) ‐ Infection (8) ‐ Peri-prosthetic fracture (4) ‐ Aseptic loosening (3) ‐ Dislocation (1) ‐ Pain (1) ‐ Malalignment (2) ‐ Other (1)
Paul Baker^1^, Rebecca Critchley, Andrew Gray, Simon Jameson, Paul Gregg, Andrew Port, David Deehan					
**Intermediate-term results of 142 single-design, rotating-hinge implants: Frequent complications may not preclude salvage of severely affected knees**	Primary (11)	‐ PJI (60) ‐ Severe arthrofibrosis requiring collateral ligament release and femoral shortening (16) ‐ Aseptic loosening (17) ‐ Instability (15) ‐ Symptomatic knee fusion requiring rotating hinge reconstruction (7) ‐ OA (11) ‐ Periprosthetic fracture (7) ‐ Mechanical failure (5) ‐ Non-union of distal femur fracture (2)	‐ KSCS 36 to 77 at the ‐ KSFS 21 to 57	Implant survival was 73%	Failed implants (39) ‐ Aseptic femoral loosening (18) ‐ Aseptic tibial loosening (1) ‐ Aseptic femoral and tibial loosening (1) ‐ Periprosthetic fracture (7) ‐ Infection (12)
Yasser R. Farid, MD, PhD^a^, Rishi Thakral, MD, FRCSI^b^, Henry A. Finn, MD, FACS	Revision (131)				
The Journal of Arthroplasty	57 months follow-up				
2015					
**Revision total knee arthroplasty for instability-outcome for different types of instability and implants**	19 revisions	‐ Instability	‐ KSFS 40 to 50 ‐ KSCS 18 to 68.5 ‐ ROM remained at 110	N/A	‐ Pain around the knee (7) ‐ No reoperations
Jaap S Luttjeboer^1^, Menno R Bénard^2^, Koen C Defoort^3^, Gijs G van Hellemondt^3^, Ate B Wymenga^3^	Followed up to 24 months postoperatively				
Journal of Arthroplasty					
2016					
**Long-term results after total knee arthroplasty with contemporary rotating-hinge prostheses**	Complex primary (74)	‐ Infection (144) ‐ Aseptic etiologies (264)	‐ KSCS improved 51 to 81 ‐ KSFS 26 to 36	Cumulative incidence of revision for any revision was 9.7% at 2 years and 22.5% at 10 years	Revisions ‐ PJI (21) ‐ Periprosthetic fracture (11) ‐ Aseptic loosening (10) ‐ Mechanical failure (10) ‐ Extensor mechanism disruption (4) ‐ Arthrofibrosis (2) ‐ Chronic patellar dislocation (1)Reoperations ‐ PJI (24) ‐ Dissociation of the prosthetic hinge (1)Intraoperative complications ‐ Fracture (20) ‐ Periprosthetic tibial fracture (9) ‐ Partial patellar tendon avulsion (2)Postoperative complications ‐ Delayed wound-healing (12) ‐ Decreased range of motion (10) ‐ Superficial wound infections (5) ‐ Periprosthetic fracture (6) ‐ Patella instability (4) ‐ Skin necrosis (3) ‐ Partial quadriceps tears (2) ‐ DVT (1) ‐ Peroneal nerve palsy (1)
Umberto Cottino^1^, Matthew P Abdel, Kevin I Perry, Kristin C Mara, David G Lewallen, Arlen D Hanssen	Revision (334)				
The Journal of Bone and Joint Surgery	Mean follow-up 4 years (range, 2 to 12 years)				
2017					
**Rotating hinge implants for complex primary and revision total knee arthroplasty**	Complex primary (14)	Primary: ‐ Severe deformity (7) ‐ Recurvatum (6) ‐ Charcot arthropathy (1)Revision: ‐ Severe instability (28) ‐ Periprosthetic joint infection (15) ‐ Dislocation (12) ‐ Aseptic loosening (7) ‐ Periprosthetic fracture (3)	‐ KSCS 35.7 to 66.2	70.7% at 5 years	Intraoperative ‐ Fracture (1)Postoperative ‐ Periprosthetic fracture (6) ‐ Extensor mechanism rupture (5) ‐ PJI (4) ‐ Mechanical failure of hinge (3) ‐ Arthrofibrosis (2) ‐ Peroneal nerve palsy (1) ‐ Recurvatum (1) ‐ Aseptic loosening (1) ‐ Superficial abscess (1)
Sean M Kearns^1^, Brian M Culp^1^, Daniel D Bohl^1^, Scott M Sporer^1^, Craig J Della Valle^1^, Brett R Levine^1^	Revision (65)				
The Journal of Arthroplasty	Minimum 2 year follow-up, mean 55.2 months				
2018					
**Rotating-hinge knee prosthesis as a viable option in primary surgery: Literature review & meta-analysis**	Primary non tumour cases (1425)	Primary non-tumour cases: ‐ Osteoarthritis ‐ Rheumatoid arthritis ‐ Post traumatic arthritis ‐ Supracondylar malunion ‐ Charcot arthropathy	Non-tumour: ‐ KSCS 84.9 ‐ KSFS 67.9Tumour cases: ‐ MSTS mean 79%	Primary non-tumour: overall survival rates for 0–5, 6–10, and 11–15 year follow-up were 92%, 82%, and 88% respectivelyPrimary tumour cases: overall survival rates for 0–5 and 6–10 year follow-up were 77% and 69% respectively	Causes of failure in primary non-tumour ‐ Infection (23) ‐ Aseptic loosening (13) ‐ Dislocation (13) ‐ Periprosthetic fracture (8) ‐ Rotation failure (5) ‐ Other (11)Causes of failure in primary tumour ‐ Infection (65) ‐ Aseptic loosening (41) ‐ Tumour recurrence (40) ‐ Fracture (26) ‐ Dislocation (1)
Ali Abdulkarim^1^, Anna Keane^2^, Shu Yang Hu^2^, Lachlan Glen^2^, Dermot J Murphy^3^	Primary tumour (936)				
Orthopaedics & Traumatology: Surgery & Research					
2019					
**Primary constrained and hinged total knee arthroplasty: 2and 5-year revision risk compared with unconstrained total knee arthroplasty: A report on 401 cases from the Norwegian Arthroplasty Register 1994–2017**	197 RHTKA cases	‐ Primary OA (64) ‐ Inflammatory arthritis (15) ‐ Post-traumatic arthritis (28) ‐ Postligament injury (37) ‐ Post infection arthritis (9) ‐ Instability (13) ‐ Neuro sequelae (10) ‐ Other (20)	N/A	2- and 5-year survival free of all revisions for primary hinged TKA was 91.0%	22 revisions ‐ Infection (16)7 perioperative complications ‐ Fracture (4)
Mona Badawy^1^, Anne Marie Fenstad^2^, Ove Furnes	Maximum 20 year follow-up				
Acta Orthopaedica					
2019					
**Survivorship of highly constrained prostheses in primary and revision total knee arthroplasty: Analysis of 6070 cases**	1033 primary	N/A	N/A	Primary: CPR of 15.9% at 11 yearsRevision: CPR at 9 years 17.5%	Primary ‐ Infection (27) ‐ Loosening (10) ‐ Fracture (14) ‐ Instability (1) ‐ Patellofemoral pain (5) ‐ Bearing dislocation (3) ‐ Patella erosion (3) ‐ Pain (3) ‐ Other (9)Revision ‐ Infection (32) ‐ Loosening (15) ‐ Instability (2) ‐ Pain (3) ‐ Arthrofibrosis (1) ‐ Fracture (8) ‐ Patellofemoral pain (3) ‐ Implant breakage (6) ‐ Other (7)
Matthew Knight^1^, Peter Lewis^1^, Yi Peng^1^, Alesha B Hatton^1^, Ben Parkinson	807 revisions				
ANZ Journal of Surgery	Mean follow-up 3.9 years for primary, 3.4 years for revision				
2020					
**Increased constraint of rotating hinge knee prosthesis is associated with poorer clinical outcomes as compared to constrained condylar knee prosthesis in total knee arthroplasty**	Primary (8)	‐ Global instability and recurvatum during primary TKA (8) ‐ Failed TKA with extensive bone loss, osteolysis and instability (17) ‐ Periprosthetic fracture (4) ‐ PJI, instability and extensive bone loss (10)	‐ ROM improvement 90.4 to 103.2 ‐ KSFS 30.7 to 43.9 ‐ KSCS 28.5 to 76.5 ‐ OKS 42.7 to 27.9	NA	‐ I + D for superficial abscess (3) ‐ PJI managed with 2 stage revision (5) ‐ Extensor mechanism defect requiring reoperation (1)
Jason Beng Teck Lim^12^, Hee Nee Pang^3^, Keng Jin Darren Tay^3^, Shi-Lu Chia^3^, Ngai Nung Lo^3^, Seng Jin Yeo	Revision (31)				
European Journal of Orthopaedic Surgery & Traumatology	Mean follow-up 4.8 years (range 2–9.2 years)				
2020					
**Guided-motion hinged knee replacement prosthesis: Early survival rate and postoperative patient function and satisfaction**	12 primary	Primary: ‐ MCL incompetence (12)Revision cases ‐ Instability (17) ‐ Infection (4) ‐ Aseptic loosening (4) ‐ Combined instability and aseptic loosening (2)	Oxford-12 Knee Score 12.9 to 27.8 at 2 year follow-up	2-year survivorship of 90.7%	Reoperations for failure of components ‐ Patella dislocation/subluxation (2) ‐ Infection (1)Reoperations with no components revised ‐ Medial capsule repair (1) ‐ Extensor mechanism repair (2) ‐ Periprosthetic fracture (1)
David L Perrin^1^, Thomas R Turgeon^1^	27 revision				
Canadian Journal of Surgery	Mean follow-up of 29.1 months				
2020					
**Periprosthetic knee infection reconstruction with a hinged prosthesis: Implant survival and risk factors for treatment failure**	39 revisions	Primary TKA PJI along with the Anderson Orthopaedic Research Institute (AORI) classification type II or III bone and ligament deficiency	‐ KSCS 37.9 points to 70.7 ‐ KSFS 25.7 to 56.5	Survivorship of 86.2% at 2 years and 70.2% at 5 years.	‐ Recurrent PJI (9) ‐ Aseptic loosening (1)
Michael Jian-Wen Chen^a^, Jui-Fu Hung^b^, Chih-Hsiang Chang^a,b^, Sheng-Hsun Lee^a,b^, Hsin-Nung Shih^a,b^, Yu-Han Chang	Mean follow-up of months 64				
The Knee					
2020					
**Management of gonarthrosis with a rotating hinge prosthesis: Minimum 10-year follow-up**	Primary (238)	‐ Advanced deformity (150) ‐ Significant bone loss (48) ‐ Ligamentous laxity (40)	‐ Mean flexion at final review 118°	Overall survivorship at a mean of 13.5 years of follow-up was 88%	19 revisions with prosthesis exchange ‐ Patella resurfacing (6) ‐ PJI (5) ‐ Mechanical failure (5) ‐ Aseptic loosening (1) ‐ Extensor mechanism disruption (1) ‐ Periprosthetic fracture (1)9 reoperations with no revision of components ‐ Patella debridement (3) ‐ Lateral release _ medial plication (2) ‐ Excision of inferior pole of patella (2) ‐ Open arthrolysis (1) ‐ Fixation of distal femur fracture (1)
Daniel Kendoff^*^, Carl Haasper^*^, Thorsten Gehrke^*^, Wolfgang Klauser^*^, Nemandra Sandiford^*^	At final review (160)				
Clinics in Orthopaedic Surgery	Minimum of 10 year follow-up, mean of 13.5 years				
2020					
**Complications and failures of non-tumoral hinged total knee arthroplasty in primary and aseptic revision surgery: A review of 290 cases**	290 cases	Primary surgery (111) ‐ Large deformity (61) ‐ Arthropathy (29) ‐ Ligament laxity (11) ‐ Primary OA (10)Aseptic revision surgery (127 patients) ‐ Loosening (75) ‐ Ligament laxity (50) ‐ Other (2)Surgery following a recent (< 3 months) fracture (52) ‐ Post traumatic sequelae (25) ‐ Fracture in an older adult (17) ‐ Non-union (8) ‐ Laxity (2)	N/A	N/A	Complications (108) ‐ Stiffness (41) ‐ Pain (37) ‐ Infection (32) ‐ Loosening (23) ‐ Extensor mechanism instability/rupture (19) ‐ Periprosthetic fracture (9) ‐ Common peroneal nerve deficit (2) ‐ Mechanical failure (2) ‐ Death(40)Revisions for Infection (31) ‐ Implant changes (12) ‐ Above knee amputation (4) ‐ Arthrodesis (2)Revisions for aseptic loosening (17)Revision for periprosthetic fracture or mechanical implant failure (11)Revisions for patellofemoral instability (2)Reoperations ‐ Arthrolysis (2) ‐ Lavage of joint for infection (13) ‐ Extensor mechanism rupture (4)
Étienne Caron^1^, Antoine Gabrion^2^, Matthieu Ehlinger^3^, Nicolas Verdier^4^, Brice Rubens-Duval^5^, Thomas Neri^6^, Pierre Martz^7^, Sophie Putman^8^, Gilles Pasquier^8^	Minimum follow-up of 5 years				
French Society of Orthopedic Surgery and Traumatology (SOFCOT)	Mean follow-up 71 months				
2021					
**Constraint in complex primary total knee arthroplasty: Rotating hinge versus condylar constrained implants**	703 primary complex non-oncological cases	NA	NA	10-year implant survival rates of 91.9%	PJI was the most common reason for revision in the two cohorts, followed by aseptic loosening.Breakage in 3 cases
Francesco Castagnini^12^, Barbara Bordini^3^, Monica Cosentino^3^, Cristina Ancarani^3^, Stefano Lucchini^4^, Giovanni Bracci^4^, Francesco Traina	Mean follow-up 6.8 years				
Archives of Orthopaedic and Trauma Surgery					
2022					
**Shortto midterm outcomes of a novel guided-motion rotational hinged total knee arthroplasty**	18 complex primaries	NA	Improved ROM on average 9 degrees	Survivorship analysis for implant revision at 2and 5-year follow-up showed a survival rate of 100% and 98.5%	Aseptic reoperations (14) ‐ Patellar Complications (7) ‐ Instability: (5) ‐ Tuberosity Fixation Issues: (1) ‐ Extensor Mechanism Failure (1)Infections (15)Implant revisions (2) ‐ Infection (1) ‐ Aseptic loosening (1)
David Yeroushalmi^1^, Simon Van Laarhoven^2^, Alex Tang^1^, Petra J C Heesterbeek^2^, Gijs Van Hellemondt^2^, Ran Schwarzkopf	129 revisions identified				
Journal of Knee Surgery	Average follow-up duration of 3.8 years (Minimum 2 years)				
2022					
**Implant survivorship, functional outcomes and complications with the use of rotating hinge knee implants: A systematic review**	568 primary	Primary ‐ Osteoarthritis ‐ Rheumatoid arthritisRevision ‐ Aseptic loosening ‐ Infection ‐ Instability ‐ Implant wear/breakage ‐ Bone loss ‐ Periprosthetic fracture	Post op ROM median was 110°KSCS 7.4 to 86.2KSFS 27.9 to 58.4OKS 11.6 to 31.5	Median survival at 1 year was 93.4% (two studies), at 5 years was 85.9% (one study) and at 10 years was 87% (three studies	NA
Joshua Xu^1,2,✉^, Lennart von Fritsch^2^, Shiraz A. Sabah^2^, Andrew J. Price^1,2^, Abtin Alvand^1,2^	413 revision				
Knee Surgery and Related Research	Median follow-up of 79.5 months				
2022					
**Rotating hinge prosthesis for primary and revision knee arthroplasty: Comparison and indications**	98 primary	Primary: ‐ Varus/valgus malalignment ‐ Instability ‐ Extension deficit ‐ Obesity ‐ Posttraumtic gonarthrosis ‐ Rheumatism ‐ Unconstrained knee cannot be balanced ‐ Bone loss ‐ OtherRevision ‐ Instability ‐ Aseptic loosening ‐ Periprosthetic fracture ‐ Arthrofibrosis ‐ Other	Primary: ‐ ROM 108.7 to 117.9 ‐ OKS: 24.4 to 8.5 ‐ KSFS 57.3 to 73.5 ‐ KSCS 35 to 89.5Revision: ‐ ROM 110.7 to 114.6 ‐ OKS 23.8 to 13.9 ‐ KSFS 56.7 to 67.8 ‐ KSCS 37.5 to 78.1	Primary: 91% at 5 yearsRevision: 70% at 5 years	Primary: ‐ Infection (7) ‐ Periprosthetic fracture (6) ‐ Aseptic loosening and instability (2)Revision: ‐ Infection (6) ‐ Aseptic loosening (6) ‐ Poor function (1)
Hans-Joachim Neuhaus, Kristin Maier	22 revision				
Biochem Research International	5 year follow-up				
2022					
**Acceptable migration of a fully cemented rotating hinge-type knee revision system measured in 20 patients with model-based RSA with a 2-year follow-up**	17 revisions	N/A	‐ KSCS 52 to 65 ‐ KSFS functional 55 to 50 ‐ OKS total 21 to 31	100% survivability at 2 years follow-up	11 complicatons ‐ Recurrence of arthrofibrosis [4] ‐ Neuropathic pain [5] ‐ Deep venous thrombosis [1] ‐ Recurrence of quadriceps tendon rupture [1] ‐ Pneumonia [1] ‐ Extended wound leakage [1] ‐ 1 patient requiring reoperation (full allograft extensor mechanism reconstruction)
Simon N. van Laarhoven^1,✉^, Malou E.M. Te Molder^2^, Gijs G. Van Hellemondt^1^, Petra J.C. Heesterbeek^2^	Follow-up 24 months				
Acta Orthopaedica					
2023					
**Condylar constrained and rotating hinged implants in revision knee arthroplasty show similar survivorship and clinical outcome: A systematic review and meta-analysis**	1131 revisions	‐ Aseptic loosening (354) ‐ Instability (272) ‐ Infection (235) ‐ Malalignment (26) ‐ Fracture (54) ‐ Arthrofibrosis (76)	KSCS of 35.1 to 80.2KSFS of 30 to 58.5ROM 69.1 to 104.1°	Survivorship of 91.6% at 5 years	Revisions ‐ Aseptic loosening (246) ‐ Instability (7) ‐ PJI (33) ‐ Fracture (5) ‐ Extensor mechanism failure (1) ‐ Knee dislocation (4) ‐ Arthrodesis (2)Reoperations ‐ MUA (5) ‐ Extensor mechanism repair (11) ‐ DAIR (8) ‐ Fracture (8)
Lenka Stroobant^1^, Thijmen de Taeye^2^, Paul Byttebier^3^, Stefaan Van Onsem^4^, Ewoud Jacobs^5^, Arne Burssens^2^, Jan Victor^2^	Mean follow-up 83.2 months				
Knee Surgery Sports Traumatology Arthroscopy					
2023					
**Risk factors for revision surgery following revision total knee arthroplasty using a hinged knee prosthesis for septic and aseptic indications**	150 revisions	‐ Infection (85) ‐ Aseptic revision (65)	NA	Survivorship for all-cause revision surgery in the aseptic cohort was 86.2% at 1 year, 81.5% at 2 years, and 71.4% at 5 yearssurvivorship for revision surgery in the septic cohort was 90.6% at 1 year, 76.5% at 2 years, and 46.1% at 5 years	Re-revision in septic cases (40) ‐ Persistent infection (22) ‐ Mechanical loosening (7) ‐ Extensor mechanism rupture (5) ‐ Periprosthetic fracture (3) ‐ Wound complications (1) ‐ Haematoma (2)Re revision in aseptic cases (16) ‐ Persistent infection (4) ‐ Mechanical loosening (3) ‐ Instability (1) ‐ Extensor mechanism rupture (2) ‐ Periprosthetic fracture (1) ‐ Wound complications (1) ‐ Haematoma (1) ‐ Non union (1) ‐ Persistent knee pain (1)
Cody C. Green^1^, John W. Stelzer, Matthew S. Kerr, Alex Tang, Luke G. Menken, Filippo Romanelli, Justin M. Miller, Frank A. Liporace, George J. Haidukewych, Richard S. Yoon	Minimum follow-up of 2 years				
Journal of the American Academy of Orthopaedic Surgeons	Average follow-up 4.9 in the septic cohort vs. 4.6 years in the aseptic cohort				
2023					
**Clinical outcomes and infection rates following revision total knee arthroplasty: Aseptic failure versus septic failure**	68 revisions for aseptic failure	Aseptic ‐ 57 patients underwent surgery due to aseptic loosening ‐ 9 due to instability ‐ 2 due to stiffness	Functional scores of all cases ‐ ROM 110 to 120 ‐ WOMAC 49.9 to 9.6 ‐ KSKS 49.4 to 86.1 ‐ KSFS 40.8 to 69	NA	Aseptic group complications ‐ Infection (3) ‐ Polyethylene change due to instability (1)Septic group complications ‐ Infection (4)
Sung-Sahn Lee^1^, Il Su Kim^2^, Young-Wan Moon^2^	26 revisions for septic failure				
Clinics in Orthopaedic Surgery	Mean follow-up durations in the aseptic and septic groups were 44.4 and 54.8 months, respectively.				
2023					

Bistolfi et al. [13] reviewed outcomes in 29 patients undergoing revision TKA using the NexGen RHTKA (Zimmer, Warsaw, Indiana). The authors reported improvements in the Hospital for Special Surgery knee score (HSS) (65.6–88.4) and ROM (90.9°–114.4°) at a mean 5-year follow-up. In another study, Perrin et al. [14] reviewed the outcomes of the Legion HK RHTKA (Smith and Nephew, Canada) in 12 patients undergoing primary TKA and 27 patients undergoing revision TKA. The authors reported improvements in functional and clinical scores at two years of follow-up. Statistically significant improvements in the OKS occurred within the first six months following surgery (16.9–28.1 *p* = < 0.001), with no further improvements occurring between 6 months and 2 years following surgery. A meta-analysis reviewing outcomes of the hinged prosthesis in primary TKA from seven studies found the mean improvement in the kKSS was 39.7 while the mean improvement in the fKSS was 40.2 at 2-to-15-year follow-up [15]. Several other studies have shown improvements in functional scores using hinged components in revision TKA for septic cases [16, 17]. The functional outcome scores in patients undergoing revision TKA for periprosthetic joint infection (PJI) were inferior compared with those undergoing revision for aseptic loosening or instability [18]. This inferiority may be related to the inevitable decrease in ROM with two-stage surgery in septic revision, and septic revision is indirectly related to larger bone defects due to failure to control persistent infection [19, 20].

Lim et al. [21] compared outcomes in 39 patients receiving condylar-constrained TKA implants (CCTKA) against 78 patients receiving RHTKA matched for gender, age, body mass index (BMI) and pre-operative clinical scores. The authors found that the CCTKA had significantly better post-operative fKSS scores (60.6 vs. 36.9 respectively, *p* < 0.001) and OKSs (23.0 vs. 29.1 respectively, *p* = 0.01) compared to the RHTKA group at two years follow-up. They also found that patients who received CCTKA were more satisfied with their surgery and felt their expectations had been met. These findings are supported by other studies comparing CCTKA with RHTKA implants [22, 23]. The authors speculated in the discussion that the more favourable outcomes they found of CCTKA implants might be due to the more extensive pathology that is generally an indication for RHTKA, with worse pre-operative pain and function scores due to this pathology. They also state that the difference in outcomes may be due to the non-standardised indications and the differing management of revision TKA between surgeons in terms of bone loss and soft tissue integrity. Finally, they hypothesised that the difference in kinematics between the RHK and CCK implants might be a further reason for the observed difference. However, further biomechanical studies will be needed to evaluate this.

The findings of these studies must be interpreted with caution as preoperative bone loss and ligamentous instability were not comprehensively matched between the treatment groups, and the outcomes may be influenced by potential confounders, such as a history of PJI, ambulation status and medical comorbidities and initial indication for receiving the specific type of implant.

## Component migration

Achieving stable component fixation in TKA can be challenging owing to loss of native bone stock, pre-existing comorbidities such as osteoporosis, and multiple previous procedures. A concern with the RHTKA design is the sheer stress at the bone-implant interface leading to an increased risk of premature component loosening and implant failure. Radiostereometric analysis (RSA) may be used to assess component micromotion and subsequent risk of component loosening following TKA [24]. Laarhoven et al. [25] used RSA to assess component migration in 20 patients undergoing TKA with fully cemented RHTKA components. The authors reported that micromotion mainly occurred within the first six weeks following surgery and predominantly affected the femoral component of the TKA. None of the study patients had failures or further component loosening at two-year follow-up, suggesting that the early micromotion of the femoral component stabilised after the initial six-week post-operative period. The early micromotion in the femoral component may be attributed to increased torsional stressors on the femoral component, which may be related to the natural deviation of the femoral autonomic-mechanical axis. As previously mentioned by Farid et al. [26] design evolution should focus on the femoral component over the more stable tibial component. A separate study assessed outcomes using fully cemented hinged components in 147 patients undergoing primary or revision TKA and found patients had only femoral component loosening at 3.8 years follow-up [10]. Further clinical trials assessing the clinical significance of these findings are needed to establish the safe thresholds for early femoral component micromotion and the long-term impact on component survivorship.

There is limited data surrounding patient factors and resultant component migration, especially within the RHTKA cohort. Two recent narrative reviews analysing component migration in TKA described that component migration might be attributed to low bone mineral density (BMD), with BMD known to also be related to age and gender [27, 28]. A post-mortem study by Howard et al. [29] looking at the contact fraction of various implant designs found that increasing age significantly impacted the fixation of the TKA components, with a reduction in contact fraction seen in the donors of higher age. However, there is a paucity of literature surrounding this topic, and further research, as well as correlation with hinged knee replacements is required.

## Biomechanical evaluations

Conventional (bi-axial) and rotating (spherical central axis) hinge constructs vary in their stresses at the bone-implant interface. The bi-axial system only has flexion-extension and internal-external rotation motion, which transmits shear forces mainly to the bushing during the movement phases of the gait cycle [30]. The spherical axial system allows for varus-valgus, internal-external, and anteroposterior motion. In knee models with deficient medial and lateral collateral ligaments, the von Mises and shear stresses are lower in rotational hinged components compared with more constrained hinged implants [31]. Furthermore, rotational hinged implants have more uniform stress distribution on the polyethene liner, which may reduce bearing surface wear over time [30, 31]. The contact stress pattern seen in the spherical central axial system has been shown to be similar to non-constrained implant designs in TKA [30, 32].

The native femoral rollback is neutralised in hinged knee constructs, leading to less posterior motion than within the native knee joint or posterior stabilised designs for TKA. In addition, the hinge implant has rotation occurring at the centre of the hinge rather than in the medial tibial plateau, as observed in the native joint. In combination, these features lead to ventral prominence of the femoral axis relative to the native tibial axis and limited femoral rollback in flexion, which may lead to increased anterior knee pain and excessive patella pressures compared with the native knee and uncoupled implants in TKA [33].

Stem design plays a pivotal role in the stability of the construct. Biomechanical studies have demonstrated similar pull-out forces for both cemented long and short stems for small bone defects, and greater pull-out forces for cemented stems compared with uncemented stems [34]. For large bone defects, long uncemented stems and short cemented stems were found to have similar pull-out strengths [35]. The central rotational stem, being either cylindrical or conical in design, dictates how much distraction can occur before stem dislocation. Cylindrical central rotational stems are superior to conical stems, and implants with short stems of cylindrical design require greater distraction forces to dislocate compared with long-stem conical designs. These cylindrical central rotational stems were found to have lower angular laxity at any amount of distraction [34]. Other authors have reported no difference in the failure rates between conical and cylindrical stems for revision TKA and suggested that stem engagement <4 cm is associated with increased rates of aseptic loosening and tibial shaft pain [36].

## Survivorship

RHTKA has component survivorship ranging from 51% to 92.5% at 10 years postoperatively [4, 37]. In a recent meta-analysis, the average survivorship for RHTKA was 92% and 82% at 5 and 10-year follow-ups, respectively [15]. It has been shown that the survivorship of primary RHTKA is higher in patients over the age of 60 years compared with those less than 60 years (94% vs. 77%) [38, 39] and patients with preoperative valgus deformities may have inferior survivorship compared to those with preoperative varus deformities (79% vs. 96%) [38]. The survivorship of RHTKA is correlated with the original indication for requiring an RHTKA, with the prosthesis rate of survival in the tumour patients being inferior to that of non-tumour patients (77% at 5 years and 69% at 10 years) [15]. This is primarily due to the recurrence of the tumour [15]. Baker et al. found that at the 5-year follow-up, there was no difference in survival when looking at the original indication of why the hinged implant was used as a replacement in the primary setting, comparing osteoarthritis with other inflammatory arthropathies and post-traumatic arthritis [40].

A systematic review by Knight et al. [41] found that for all age groups except those over 75, the survivorship of hinged versus fixed stabilised TKA in the primary setting was comparable. The patients over 75 showed higher revision rates with hinged implants secondary to periprosthetic fractures, possibly related to higher rates of osteoporosis in the elderly and higher implant-to-bone stress at the stem. However, they could not comment on the significance of this. They found that when the indication for primary TKA was osteoarthritis, there were no significant differences in survivorship for these patients, however for the indication of periarticular fracture, the hinged knee outperformed the fixed stabilized TKA, with a cumulative percent revision rate of 0.9% versus 3.4% for that of the fixed stabilised TKA. The authors attributed this to the lack of collateral ligament support in these fractures, and the constrained nature of the hinged implant being able to accommodate the instability created by this loss.

A meta-analysis by Yoon et al. [23] included outcomes from 12 studies reporting on revision TKA and found that RHTKA had superior survivorship compared with CCTKA (87.4% vs 75%) at short-term (<5 years) follow-up, but comparable outcomes (81.3% vs 83.8%) were found between the two groups at mid-term (5–10 years) follow-up. The increased constraint in the RHTKA group may improve early implant stability and survivorship. However, the increased sheer forces at the bone-implant interface may lead to increased aseptic loosening with longer-term follow-up [1]. Badawy et al. [42] reviewed outcomes from the Norwegian Arthroplasty register and reported that CCTKA had improved survivorship compared with RHTKA at two- and five-year follow-ups. Importantly, when infection as the reason for the revision was excluded, the RHTKA, CCTKA and conventional TKAs had similar survivorships of 96% at 5 years follow-up [42]. Similarly, Stroobant et al. [11] conducted a meta-analysis using 40 studies and found no differences in survivorship between RHTKA (91%) and CCTKA (89%) at five-year follow-ups.

A systemic review by Xu et al. in 2022 [43] reviewed outcomes for hinged implants in both primary and revision TKA. For primary TKA with hinged implants, the median survivorship was 93.4% at one year and 85.9% at five years. For revision, TKA, the median survivorship was 79.6% at one year, 77% at five years and 65.1% at 10 years follow-up [43]. Perrin et al. [14] reported 100% survivorship for primary TKAs with hinged implants at one and two years of follow-up, with 91% and 85.9% survivorship for revision TKAs with hinged implants at one and two years, respectively. The inferior survivorship for revision TKA is likely related to pre-existing factors, including the indication as to why the patient requires revision, such as septic or aseptic loosening, bone loss and reduced bone quality. Chen et al. [16] reviewed outcomes specifically in patients undergoing revision TKA with hinged implants for PJI and reported survivorship of 86% at two years and 70% at five-year follow-up. The main reasons for failure requiring revision surgery were recurrence of the infection and aseptic loosening. Other studies reviewing outcomes in both primary and revision hinge replacement have reported overall survivorship of 89–93% at two years and 79% at five years follow-up [13, 44].

## Complications

Despite improvements in the design and materials used in hinged implants for TKAs, the overall complication rates associated with their use range from 9.2% to 63% [45]. Higher rates of complications have been reported in patients undergoing TKAs with hinged implants for revision surgery compared with primary surgery. Of note, most complications with hinged implants in revision TKA occur within two years post-operatively [7, 45, 46], and the most commonly reported complications include aseptic loosening, stiffness, periprosthetic fractures, PJI, stiffness and extensor mechanism failure [14, 47]. Less common complications include patellar instability and prosthetic dislocation (0.7–4.4%) [48]. These complications may reflect a selection bias as patients undergoing hinged knee implants may be elderly with multiple comorbidities, poor physiological reserve and have had multiple previous knee procedures. Therefore, the additional risk of these complications due to the implant design is challenging to quantify.

In the primary setting, PJI (11%) remains the most common mode of failure. Aseptic loosening tends to affect the femoral implant more than the tibial implant, with an increased risk of loosening in patients who are younger at the time of replacement or undergoing revision surgery [47]. As expected, high stress at the bone-cement interface and osteolysis of the polyethylene liner are the main causes of aseptic loosening [49]. When periprosthetic fractures occurred, this was most commonly found in the femur [47].

An important cause of failure in revision RHTKA is PJI, particularly following one or two-stage revision surgery for a previous PJI. Chen et al. [16] reviewed outcomes in 58 patients undergoing second-stage revision TKA with hinged implants for PJI and reported infection-free survival was 68.9% at two years and 60.6% at five-year follow-ups. The multi-variate analysis found that obesity (HR = 3.11), high-virulence pathogens (HR = 3.44) and polymicrobial infection (HR = 3.59) were independent risk factors for PJI. Green et al. [50] reviewed outcomes in 85 patients undergoing revision RHTKA with previous infection against 65 patients undergoing revision RHTKA for aseptic reasons. The study found that the septic group had an increase in return to the operating room (46% versus 25%, *p* = 0.01), with revision-free survival improved in the aseptic group. RHTKA implants that were associated with concomitant flap reconstruction had an even higher risk for requiring revision surgery, with a 3-fold increase noted in Green’s study using regression analysis (*p* < 0.0001). A further complication noted in RHTKA was dislocation. Dislocation of the implant may occur if the flexion-extension gaps are not balanced, though this is becoming less common with modern hinged implant designs that have an anti-dislocation mechanism [2, 44, 47, 51]. Pre-operative workup is essential for patient optimisation to decrease the risk of PJI, including nutritional support and weight control, smoking cessation and multi-resistant organism eradication.

## Conclusion

RHTKA remains an essential part of the armamentarium in primary and revision cases with poor bone stock, soft tissue compromise, gross instability, and periprosthetic fractures. Studies have shown that an RHTKA may be used in both primary and revision scenarios to improve the ROM and functional outcomes. RSA has shown that some RHTKA designs are associated with early femoral component micromotion, but this has not translated to increased failure or revision rates. RHTKA designs may transfer the centre of rotation anteriorly, leading to increased anterior knee pain and abnormal patella pressures. Implant survivorship with modern rotating hinge implant designs is comparable to CCTKA at mid-term follow-up. The most common complications with an RHTKA include aseptic loosening, PJI, stiffness, and fractures. There is a paucity of evidence in regards to key factors such as the activity level of the patient and how this may influence the results of RHTKA, the difference in cost between prostheses and how this may influence the implant of choice, variations in rehabilitation protocols for RHTKA compared to others, time required for return to work or sport following RHTKA, or differences in surgical technique which may influence a surgeon’s predilection for one type of prosthesis over another. These may be areas of research in the future.

## Data Availability

The research data associated with this article are included within the article.
